# *Helicobacter species *are associated with possible increase in risk of biliary lithiasis and benign biliary diseases

**DOI:** 10.1186/1477-7819-5-94

**Published:** 2007-08-20

**Authors:** Manoj Pandey

**Affiliations:** 1 Department of Surgical Oncology, Institute of Medical Sciences, Banaras Hindu University, Varanasi 221 005, India

## Abstract

**Background:**

Hepato-biliary tract lithiasis is common and present either as pain or as asymptomatic on abdominal ultrasonography for other causes. Although the DNA of *Helicobacter *species are identified in the gallbladder bile, tissue or stones analyzed from these cases, still a causal relationship could not be established due to different results from different geographical parts.

**Methods:**

A detailed search of pubmed and pubmedcentral was carried out with key words *Helicobacter *and gallbladder, gallstones, hepaticolithiasis, cholelithiasis and choledocholithiasis, benign biliary diseases, liver diseases. The data was entered in a data base and meta analysis was carried out. The analysis was carried out using odds ratio and a fixed effect model, 95% confidence intervals for odds ratio was calculated. Chi square test for heterogeneity was employed. The overall effect was calculated using Z test.

**Results:**

A total of 12 articles were identified. One study used IgG for diagnosis while others used the PCR for Ure A gene, 16 S RNA or Cag A genes. A couple of studies used culture or histopathology besides the PCR. The cumulative results show a higher association of *Helicobacter *with chronic liver diseases (30.48%), and stone diseases (42.96%)(OR 1.77 95% CI 1.2–2.58; Z = 2.94, p = 0.003), the effect of each could not be identified as it was difficult to isolate the effect of helicobacter due to mixing of cases in each study.

**Conclusion:**

The results of present meta analysis shows that there is a slight higher risk of cholelithiasis and benign liver disease (OR 1.77), however due to inherent inability to isolate the effect of stone disease from that of other benign lesions it is not possible to say for sure that *Helicobacter *has a casual relationship with benign biliary disease or stone disease or both.

## Background

The benign diseases of the hepatobiliary system and the stone disease is one of the common problems encountered in some parts of the world. Some of the causes of biliary lithiasis are known while others are still being debated. Infection of the biliary tract has also been suspected to lead to stone formation, which is also been described as tomb of a bacteria. There is an increasing concern that preexisting untreated stone disease could lead to malignancy in long run [1].

Although *Helicobacter *species are identified in the bile, tissue and stones of the patients with stone disease and the benign biliary diseases, due to differing results that have been obtained from different geographical regions no causative relationship could be established till date [2]. This article systematically reviews the evidence available on the role of *Helicobacter *species in benign diseases and biliary tract lithiasis and presents a meta analysis of results.

## Methods

A detailed pubmed search was made using key words 'hepatobiliary', 'biliary','gallbladder' and 'neoplasia', or 'tumor', stone, lithiasis, cholelithiasis, hepaticolithiasis, choledocholithiasis, gallstone and *Helicobacter*. The search was then limited to humans. The articles were carefully read and were classified into observational or case-control study type. The data on method of detection, number of positive cases for *Helicobacter *in subjects and controls and the type of organisms identified were extracted and entered in the database prepared for the purpose. Total samples analyzed in the study were taken into account irrespective of methods of detection used. If a study used three methods of detection, the sample of all the three methods was summed to get total number and number of positive cases. No attempt was made to study the effect of method of detection in the present analysis. Meta analysis was carried out for the above data from the case control studies despite their heterogeneity with the goal that if an association is observed in this meta analysis this will help in designing further studies and will also help in understanding what kind of studies are exactly required.

Meta analysis was carried out using odds ratio and a fixed effect model, 95% confidence intervals for odds ratio was calculated. Chi square test for heterogeneity was employed. The overall effect was calculated using Z test.

## Results

The abstract of each article was screened by author and a total of 12 articles that were found to be relevant were identified [3-14]. These articles form the basis of the present review. The salient features of these articles on methodology, diseases studied and results are summarized in table 1.

**Table 1:** Characteristics of case control studies on *Helicobacter sp *in benign hepatobiliary diseases and stone diseases.

**Reference and year**	**Method of detection**	**Specimen**	**Disease**	***Helicobacter *in subjects n/N**	***Helicobacter *in controls n/N**	**Organism identified**
Myung et al 2000 [3]	PCR (UreA) (26 kDa Ag)	Intrahepatic Bile, biliary duct tissue, gallbladder	Hepatobiliary diseases	4/43 ureA 5/43 26 kDa	0/8 0/23	H. Pylori
Nilsson et al 2000 [4]	PCR (16s rRNA)	Liver	PSC PBC	9/12 11/12	0/10 control 1/13 NCLC	H. Sp.
Harada et al 2001 [5]	PCR (16s rRNA)	Bile, biliary epithelium	Intrahepatic calculi	3/14 bile 5/8 biliary epithelium	0/9 0/7	Campylobacter sp.
Leong et al 2001 [6]	PCR (16s rRNA)	Bile	CBD stone Cholangitis	4/25	0/4	H. Sp.
Presser Silva et al 2003 [7]	Culture PCR (16s rRNA)	Bile Gallbladder tissue	Cholelithiasis	20/64 culture 28/51 histology 24/56 bile	2/18	H pylori
Chen W et al 2003 [8]	PCR	Gallbladder	Gall stones	29/60 symptomatic 6/10 asymptomatic	15/37	*Helicobacter *pylori
Vorojova T et al 2006 [9]	IgG Elisa	Serum	Chronic liver disease	13/35 7/32 1/27	4/81; 0/16 7/84; 0/6 12/89; 3/17	*H. hepaticus* *H. bilis* *H. pullorum*
Abayli et al 2005 [10]	16s rRNA PCR HPE	GB stone	Cholesterol stones	7/77	6/20	*H pylori*
Farshad Sh et al 2004 [11]	16s RNA	Stones Bile	Gall stones	6/33 stones 4/33 bile	0/40	*H pylori* *H spp*
Bulajic et al 2002 [12]	ureA gene PCR	Bile	Biliary lithiasis cholengitis	26/48 stone 9/17 cholangitis	1/7	*H Pylori*
Figura et al 1998 [13]	CagA	Bile	gallstones	92/112	90/112	*H. pylori*
Matsukura et al 2002 [14]	PCR 16s rRNA	Bile	Biliary tract cancer Gallstones	24/29 malignant 18/42 stone	4/14	*H bilis*

Chr. – chronic; CaGB – gallbladder cancer; CC – cholangiocarcinoma; HCC – hepatocellular carcinoma

PSC – Primary sclerosing cholangitis; PBC – primary biliary cirrhosis; CBD – common bile duct; GB – gallbladder

**Figure 1:**
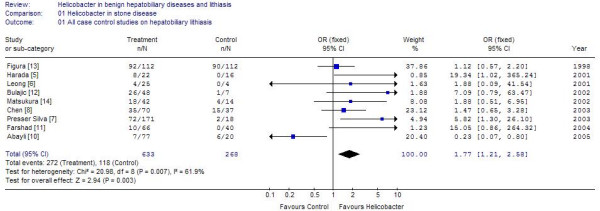
Results of Meta analysis of 9 studies on lithiasis patients as cases.

**Figure 2:**
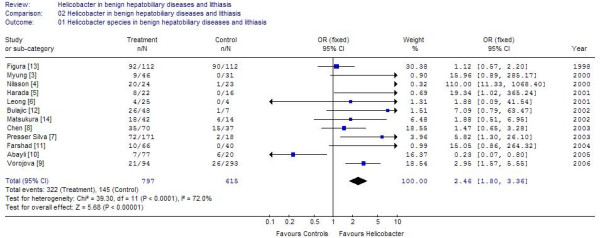
Results of Meta analysis of 12 studies on lithiasis and benign biliary diseases including chronic liver diseases.

**Figure 3:**
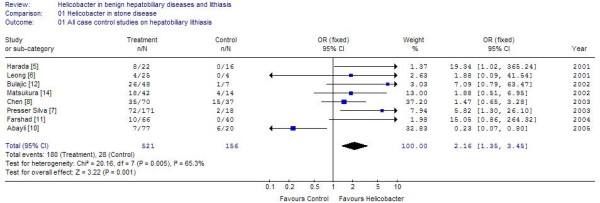
Results of meta analysis of 8 studies on lithiasis after exclusion of study of Figura et al on CAg A protein.

**Figure 4:**
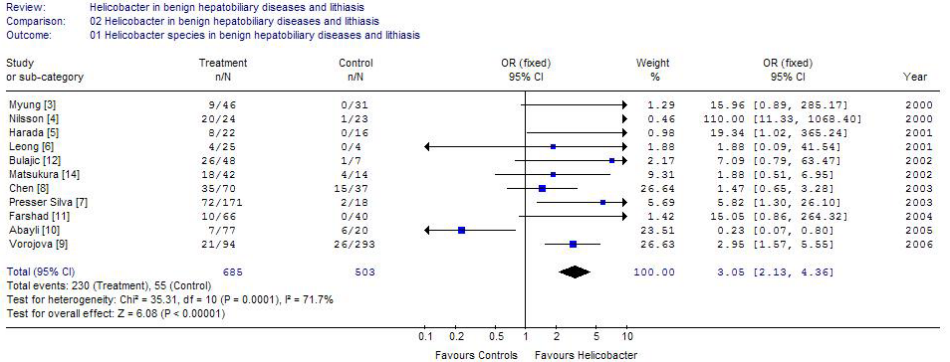
Results of meta analysis of 11 studies on lithiasis and benign biliary diseases after exclusion of study of Figura et al on CAg A protein.

Of the case control studies, 8 studies were purely on gallstone disease [5-7,10-14] (table 1; figure 1), While 4 others examined the population with stones beside either other chronic diseases or malignancies of biliary tract [3,4,8,9]. The cumulative sample size of these 12 studies was 797 of which 322 (40.4%) were positive for *Helicobacter *species. The positivity in the controls was 145/615 (23.5%). One study each used UreA gene analysis and 26 kDa antigen for identification of *Helicobacter*, another used CagA, while rest used 16s rRNA or DNA based PCR. Positivity in biliary epithelium was much higher 40–62.5% [3,5] compared to that in bile. Seven of these identified *H. pylori*, one study identified *Campylobacter sp *while two identified *H. bilis*, and rest identified *Helicobacter sp*. The positivity rate among controls was 23.5%. Of the 9 studies where the sample size of lithiasis patients was available 272/633 samples from cases and 118/268 samples from controls were positive. The cumulative odds ratio of these studies was 1.77 (95% CI 1.21–2.58) (Z = 2.94, p = 0.003). Three studies looked at the patients with benign hepato-biliary diseases. Addition of these studies raised the sample size to 797 cases and 615 controls. The odds ratio after addition of these studies increased to 2.46 (95% CI 1.8–3.36; I2 76%, Z = 5.68) (figure 2). It was found that the study of Figura *et al*., [13] used Cag A in bile and gallstones for detection of *H pylori *and contributed nearly 1/3 of the weight in the overall effect due to its large sample size. Exclusion of this study from the estimation lead to Odds of 2.16 [95% CI 1.35–3.45] in stone disease with I^2 ^of 65.3% Z value of 3.22 (p = 0.001) and odds of 3.05 [95% CI 2.13–4.36] in benign diseases including stones with I^2 ^of 71.7% and Z value of 6.08 (p < 0.0001). Thus increase in odds was observed for both groups by exclusion of this study (Figure 3 and 4).

## Discussion

The genus of *Helicobacter *has expanded to include number of species [15]. The most studied among these is *Helicobacter pylori (H. Pylori)*, that colonizes the stomach and has been found to be associated with benign diseases like gastritis [16], and peptic ulcer disease [17]. *H*. *pylori *is a spiral highly motile microaerophillic organism, uniquely adopted to survive by burrowing into the mucous layer when it is well protected from the host environment.

Some other species like *H*. *hepaticus *and *H*. *bilis *have been shown to infect the liver and the presence of them has been associated with hepatitis and hepatobiliary neoplasms [1,15,18,19].

The complete genomic sequence of *H*. *Pylori *and *H*. *hepaticus *is now known. With the identification of *H*. *pylori *the paradigm for the management of peptic ulcer, chronic gastritis, MALT lymphoma and gastric adenocarcinoma has undergone paradigm shift [20,21]. The *H pylori *infection is now also been implicated in diverse conditions like coronary artery disease, autoimmune thrombocytopenia, skin diseases, and growth retardation in children [22-25].

The *Helicobacter sp *have been reported to survive in bile juice and that has prompted people to think "could this bacterial colonization of bile be responsible for hepatobiliary disease"? *Helicobacter species *has been identified in bile juice of patients with cholelithiasis, primary sclerosing cholangitis and primary biliary cirrhosis [26,4,29].

It is also proposed that *Helicobacter *may also promote the risk of stone formation by acting as a foreign body to form a nidus around which the stone may develop or it may produce hydrolyzing enzymes or neucleating proteins like immunoglobulins. CagA protein of *Helicobacter *has been found to have a homology with aminopeptidase and hence can increase the gallstone formation. Beside these *Helicobacter sp*. Have also been proposed to increase the lithogenicity by production of soluble antigens that may bind to and inhibit key hepatobiliary genes like *muc *[31]. This may lead to modulation of enterohepatic cycling of conjugated bile acids through genetic regulation of absorption at enterocyte level or modulation of the transit time through gut [31]. Host response to *Helicobacter *antigens in form of cytokines, and other inflammatory mediators are also proposed to play a role [31]. However, the exact mechanism and exact role played by *Helicobacter *is still speculated. The results of present meta analysis show a positive association of helicobacter with benign biliary diseases and lithiasis, however further case control studies are needed to further elucidate their relationship.

## Conclusion

The results of meta analysis suggest that the *Helicobacter *may play a role however due to heterogeneity of the sample and variations in the methodology for detecting *Helicobacter *these results should be seen with skeptic view and further studies are warranted to definitely answer the question.

## Competing interests

The author(s) declare that they have no competing interests.

## Authors' contributions

MP: Conceived the idea, carried out the literature search, performed the meta analysis and wrote the manuscript.
